# The synergistic effects of applying pulsed radiofrequency lesioning of the suprascapular nerve plus physical therapy on pain and function in patients with adhesive capsulitis: A protocol of a prospective, randomized, controlled trial

**DOI:** 10.1097/MD.0000000000025431

**Published:** 2021-04-09

**Authors:** Weifeng Liao, Xinning He, Zhiyong Du, Yi Long

**Affiliations:** a Department of Spine Surgery, The Orthopedics Hospital of Traditional Chinese Medicine Zhuzhou City; b Department of Joint Surgery, Zhuzhou Central Hospital, Zhuzhou, Hunan, China.

**Keywords:** adhesive capsulitis, double-blind, physical therapy, protocol, pulsed radiofrequency, randomized, suprascapular nerve

## Abstract

**Background:**

To our knowledge, there have been no published clinical trials to assess the synergistic effects of applying pulsed radiofrequency (PRF) stimulation of the suprascapular nerve (SSN) plus physical therapy on pain and function in patients with adhesive capsulitis. Therefore, we will conduct this present randomized, double-blind study to evaluate the synergistic effects of applying PRF stimulation of the SSN plus physical therapy on pain and function in patients with adhesive capsulitis.

**Methods:**

The study protocol is a randomized, controlled, double-blind design. Recruitment will be started in March 2021 and completed in October 2022. The treating surgeon will assess 90 patients for eligibility. The study protocol was approved through Institutional Review Board in the People's Hospital of Beilun district of Ningbo. Each patient will be randomized into 3 treatment groups, receiving PRF stimulation of the SSN or physical therapy or both of them. After baseline examination, all patients will be given a full explanation of the treatment protocol and will be required to sign a written informed consent for study participation and for publication of the results. All the data collectors, surgeons, statistical analysts, as well as result assessors are not aware of grouping assignment. The outcomes include Constant score, visual analog scale score, range of motion, and strength.

**Results:**

This protocol will provide a reliable theoretical basis for the following research.

**Conclusion:**

It is assumed that there will be a remarkable difference in postoperative outcomes between the intervention and control groups.

**Trial registration number:**

10.17605/OSF.IO/PZ9ES.

## 1 Introduction

Adhesive capsulitis, or “frozen shoulder,” is a painful condition commonly encountered in outpatient orthopedic clinics. It has a prevalence of 2% to 5% among outpatients but of about 20% to 30% in patients with diabetes mellitus.^[1]^ The pathogenesis of this condition remains unclear, although associated factors include female sex, trauma, age over 40 years, diabetes mellitus, prolonged immobilization, thyroid disease, stroke, myocardial infarction, and autoimmune disease.^[2]^ Common nonoperative regimens for adhesive capsulitis include supervised neglect with analgesia, supervised physical therapy, an intra-articular corticosteroid injection, a suprascapular nerve (SSN) block, manual brisement, and saline dilatation. No therapeutic intervention is currently universally accepted as most effective for restoring range of motion and decreasing pain in patients with this disease.^[3,4]^

Recent research reported that pulsed radiofrequency (PRF) lesioning of the SSN was effective in treating chronic shoulder pain, including adhesive capsulitis, and it facilitated functional recovery without risking paralysis of the supra- or infraspinatus muscles.^[5]^ PRF is designed to create bursts of heat through the delivery of an electrical field. The short bursts allow for the alleviation of pain in neural tissue, while allowing the tissue to maintain a temperature below 42°C, preventing the nerve damage associated with conventional radiofrequency.^[6]^ Many studies have confirmed the effectiveness of PRF for multiple pain conditions.^[7]^ Recent studies have successfully applied ultrasound-guided PRF stimulation to the medial nerve (carpal tunnel syndrome), interscalene brachial plexus (neoplastic plexopathic pain), intercostal nerve, trigeminal nerve (trigeminal neuralgia), and sciatic nerve (chronic knee pain).^[8–10]^ The ultrasound-guided technique offers improved quality of regional nerve blocks and limits neural trauma. Moreover, ultrasound is radiation-free, more affordable, and more expedient than the computerized tomography and fluoroscopy that are conventionally performed for PRF guidance.

Physical therapy agents including superficial and deep heat with analgesic currents are commonly used to decrease pain and spasm owing to adhesive capsulitis. Rehabilitation methods, including stretching, range of motion, strengthening exercises, and mobilization maneuvers are used to improve joint mobility and restore function. However, these interventions are usually painful and prevent the individual from fully participating in the rehabilitation program. Therefore, PRF lesioning of the SSN may be preferred for adhesive capsulitis before applying physical therapy.^[11,12]^

To our knowledge, there have been no published clinical trial to assess the synergistic effects of applying PRF stimulation of the SSN plus physical therapy on pain and function in patients with adhesive capsulitis. Therefore, we will conduct this present randomized, double-blind study to evaluate the synergistic effects of applying PRF stimulation of the SSN plus physical therapy on pain and function in patients with adhesive capsulitis. It is assumed that there will be a remarkable difference in postoperative outcomes between the intervention and control groups.

## 2 Material and method

### 2.1 Study design

The study protocol is a randomized, controlled, double-blind design. The study protocol was approved through Institutional Review Board in the People's Hospital of Beilun district of Ningbo (HN20201208) and was registered in the OSF Registries (with number: 10.17605/OSF.IO/PZ9ES).

### 2.2 Participants

The study included patients with idiopathic adhesive capsulitis of the shoulder between the ages of 18 and 80 years who had primary adhesive capsulitis of the shoulder. Our diagnosis of adhesive capsulitis of the shoulder was based on a consensus definition, characterized by functional restriction of both active and passive shoulder motion for which radiographs of the glenohumeral joint are essentially unremarkable. All patients underwent a thorough history, physical examination, and radiographic imaging studies, including standard plain radiographs and ultrasound imaging scan by the treating orthopedic surgeon with 10-year of shoulder US experience.

The exclusion criteria for the study were patients with diagnosed rotator cuff tear, calcific tendinitis, or impingement syndrome by ultrasound imaging, patients with symptomatic cervical radiculopathy, patients with previous fracture or surgery around shoulder, acute or chronic infection around shoulder, a pregnancy, patients with a recent history of injection around the shoulder (within 6 weeks), and other secondary conditions possibly related to shoulder dysfunction (rheumatologic disease or hemiplegia).

### 2.3 Recruitment and randomization

**Figure 1:**
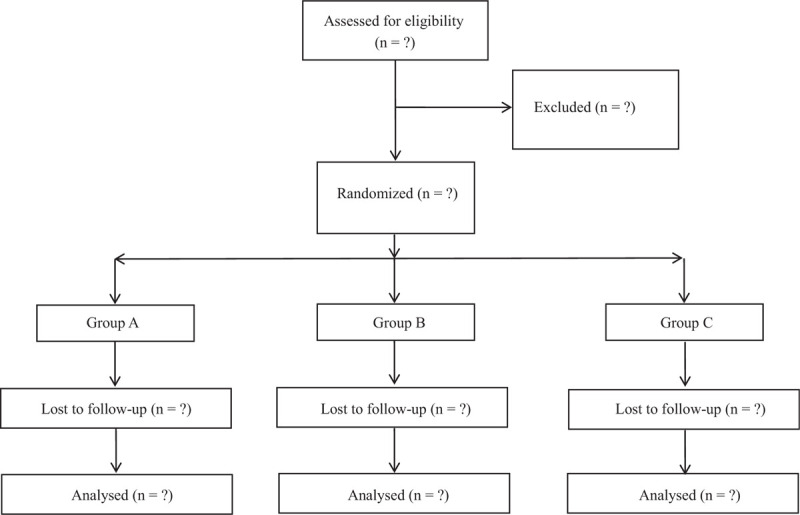
Consolidated Standards of Reporting Trials (CONSORT) diagram of patient flow through the study.

Recruitment will be started in March 2021 and completed in October 2022. The treating surgeon will assess 90 patients for eligibility. Written informed consent will be obtained from patients. Then, each patient will be randomized into 3 treatment groups, receiving PRF stimulation of the SSN or physical therapy or both of them. To allocate treatment, a randomization sequence with random permuted blocks will be generated by randomization program on internet website (www.randomizer.org). Sequentially numbered opaque sealed envelopes containing treatment allocations will be prepared and opened in sequence by an independent research assistant who is not involved in treatment. All the data collectors, surgeons, statistical analysts, as well as result assessors are not aware of grouping assignment (Fig. 1).

### 2.4 Intervention protocol

In order to perform the surgical procedure under strict sterile conditions, the PRF lesioning procedure will be performed in the operating room. Each patient is placed in a prone position, and the skin overlying the operation area is prepared and draped. A standard RF lesion generator (Neurotherm JK 25T) is used for the whole procedure. The suprascapular notch is identified under C-arm fluoroscopy at an angle slightly oblique to the treated side and angled cephalo-caudal. After sterile preparation and administration of local anesthesia, a 22-gauge 10 mm active tip RF needle 10 cm in length is inserted and advanced toward the suprascapular notch. Proper localization of nerve is achieved by sensory and motor stimulation. Sensory stimulation is performed (50 Hz; 1 ms pulsed width; up to 0.5 volt) and the patient reports paresthesia in the shoulder joint. Upon motor stimulation (2 Hz; 1 ms pulsed width; up to 1 V), muscle contractions in the suprascapular and infrascapular muscles are observed. Two PRF cycles of 180 s are performed after localization. The RF current is delivered at a width of 20 ms and at 45 V. The tip temperature works at 38°C initially, and then the temperature increases slowly to 42°C within 30 s. The patients are observed for 30 min after the PRF lesioning procedure. If no significant complications (including pain, bleeding, and pneumothorax) are observed, the patient will be discharged.

### 2.5 Physical therapy

Initially, hot pack application and transcutaneous electronic nerve stimulation are performed for groups. Then, for 12 weeks, stretching exercises, mobilization, and therapeutic exercises (30 min per session, 3 sessions per week) are performed under the guidance of a therapist who is blinded to patient allocation.

### 2.6 Outcomes parameters

All patients will be assessed for pain and functional status before and immediately after the treatment by a researcher blinded to the patient groups. The range of shoulder mobility and functional status will be assessed using the Constant Shoulder Scale, which was developed to evaluate functional recovery of the shoulder. The total Constant score ranges from 0 to 100 points, with higher scores indicative of better function. The score is divided into four sections: pain (15 points), activities of daily living (20 points), range of motion (40 points), and strength (25 points). Pain is scored using a visual analog scale. The activities of daily living score are divided into 4 subscales of sleep, work, recreational activities, and ability to position the hand in space. Range of motion is assessed in 4 active positions, with 10 points for each. Flexion and abduction are assessed by a goniometer, whereas functional internal and external rotation is assessed by the position of the hand. Strength is tested by a calibrated spring dynamometer, at 90° abduction of the shoulder with the forearm pronated and scored as the maximum of 3 repetitions (Tables 1 and 2).

**Table 1:** Patient baseline demographics.

Demographics	Combined group	PRF lesioning of the SNN group	Ultrasound group	*P*
Number of patients (shoulders)				
Age, yr				
Male sex [no. (%)]				
BMI^∗^, kg/m^2^				
Left side [no. (%)]				
Follow-up^∗^				

BMI = body mass index, PRF = pulsed radiofrequency, SSN = suprascapular nerve.

∗ The values are given as the mean and the SD.

**Table 2:** Postoperative outcomes.

Outcomes	Combined group	PRF lesioning of the SNN group	Ultrasound group	*P*
VAS				
Constant score				
Range of motion				
Muscle strength				

PRF = pulsed radiofrequency, SSN = suprascapular nerve, VAS = visual analog scale. The values are given as the mean and the SD.

### 2.7 Statistical analysis

All analyses will be performed using SPSS for Windows, version 16, and GraphPad InStat. Analysis of variance is used for comparing mean values of patient's age, weight, height, and body mass index. For nonparametric measures such as pain score and functional scores, differences between baseline and posttreatment scores for each group are computed by the Wilcoxon signed ranks test. The difference between each treatment group is performed by the Kruskal--Wallis test. The level of statistical significance is set as *P* < .05. A power analysis is conducted to estimate the requisite sample size. From preliminary research in our hospital, a clinically significant difference reduction in the pain score was defined as 3 cm; a standard deviation of 3.49, probability of a Type I error of 0.05, and power of 0.8 resulted in an estimated sample size of 25. Consequently, a total of 90 subjects will be recruited.

## 3 Discussion

In the early stages of adhesive capsulitis, the aim of management is to alleviate pain so that rehabilitation can be used to restore normal shoulder range of motion and activity. Previous studies indicated that some patients could not finish rehabilitation because of the presence of intractable pain, leading to subsequent studies for investigating the effect of pain relief before rehabilitation. One of the most frequently used methods is intraarticular injection with a steroid. However, one review concluded that an intraarticular steroid injection before rehabilitation provided only a short-term benefit. Our study is the first to investigate the effect of PRF lesioning of the SSN plus physical therapy. The rapid onset of pain relief after PRF lesioning of the SSN apparently makes it easier for patients to tolerate rehabilitation therapy. However, more research is needed before we can recommend widespread adoption of this method as a treatment for adhesive capsulitis.

To our knowledge, there have been no published clinical trial to assess the synergistic effects of applying PRF stimulation of the SSN plus physical therapy on pain and function in patients with adhesive capsulitis. Therefore, we will conduct this present randomized, double-blind study to evaluate the synergistic effects of applying PRF stimulation of the SSN plus physical therapy on pain and function in patients with adhesive capsulitis. It is assumed that there will be a remarkable difference in postoperative outcomes between the intervention and control groups.

## Author contributions

Weifeng Liao and Xinning He conceived, designed, and planed the study. Weifeng Liao and Xinning He are recruiting the study participants and performing the interventions. Zhiyong Du supervised the study. Zhiyong Du will interpret and analyze the data. Weifeng Liao drafted the manuscript. Yi Long critically revised the manuscript for important intellectual content. All authors have full access to the manuscript and take responsibility for the study design. All authors have approved the manuscript and agree with submission.

**Conceptualization:** Xinning He.

**Data curation:** Weifeng Liao.

**Formal analysis:** Weifeng Liao, Xinning He.

**Funding acquisition:** Yi Long.

**Investigation:** Weifeng Liao, Xinning He, Zhiyong Du.

**Methodology:** Weifeng Liao, Zhiyong Du.

**Project administration:** Zhiyong Du, Yi Long.

**Resources:** Yi Long.

**Software:** Xinning He.

**Supervision:** Yi Long.

**Validation:** Xinning He.

**Writing – original draft:** Weifeng Liao.

**Writing – review & editing:** Zhiyong Du, Yi Long.
